# Adapting Document Similarity Measures for Ligand-Based Virtual Screening

**DOI:** 10.3390/molecules21040476

**Published:** 2016-04-13

**Authors:** Mubarak Himmat, Naomie Salim, Mohammed Mumtaz Al-Dabbagh, Faisal Saeed, Ali Ahmed

**Affiliations:** 1 Faculty of Computing, Universiti Teknologi Malaysia, Skudai, Johor 81310, Malaysia; naomie@utm.my (N.S.); mohamad.aldabbagh@protonmail.com (M.M.A.D.); faisalsaeed@utm.my (F.S.); alikarary@gmail.com (A.A.); 2 Faculty of Engineering, Karary University, Khartoum 12304, Sudan

**Keywords:** chemoinformatics, similarly search, similarity coefficients, virtual screening, drug discovery

## Abstract

Quantifying the similarity of molecules is considered one of the major tasks in virtual screening. There are many similarity measures that have been proposed for this purpose, some of which have been derived from document and text retrieving areas as most often these similarity methods give good results in document retrieval and can achieve good results in virtual screening. In this work, we propose a similarity measure for ligand-based virtual screening, which has been derived from a text processing similarity measure. It has been adopted to be suitable for virtual screening; we called this proposed measure the Adapted Similarity Measure of Text Processing (ASMTP). For evaluating and testing the proposed ASMTP we conducted several experiments on two different benchmark datasets: the Maximum Unbiased Validation (MUV) and the MDL Drug Data Report (MDDR). The experiments have been conducted by choosing 10 reference structures from each class randomly as queries and evaluate them in the recall of cut-offs at 1% and 5%. The overall obtained results are compared with some similarity methods including the Tanimoto coefficient, which are considered to be the conventional and standard similarity coefficients for fingerprint-based similarity calculations. The achieved results show that the performance of ligand-based virtual screening is better and outperforms the Tanimoto coefficients and other methods.

## 1. Introduction

The past few years have witnessed more attention to chemoinformatics and now it has become an active multidisciplinary research area that covers wide aspects of chemistry and drug discovery using different tools and technology. Virtual screening (VS) is considered as one of the most relevant aspects of chemoinformatics; the term screening is given to the selection of molecules for bioactivity testing, and the use of computer methods for the selection of molecules is hence generally referred to as virtual screening [1]. The purpose of VS methods and techniques is to screen a large database of molecules in order to find compounds that fit some established criteria [2]. VS is now one of the important processes of discovering new ligands on the bases of biological structure and it has many definitions, one of which is: “Use of high-performance computing to analyze large databases of chemical compounds in order to identify possible drug candidates” [3]. VS is different and contrasts high-throughput screening (HTS). The experiments are not really done in a chemical laboratory, as HTS, and the compounds do not need to physically exist as they are virtually done by computers programs and methods. They actually rely on computational methods that are used to search molecular databases and identify molecular structures that are most likely to bind to a drug target, typically a protein receptor or enzyme. The VS research challenge is how to develop sophisticated techniques that can easily synthesize and analyze large molecules numbers to help in the drug discovery process [3,4,5,6,7].

For molecular data, there are several types of descriptors, where the molecular descriptor is defined as “The final result of a logical and mathematical procedure which transforms chemical information encoded within a symbolic representation of a molecule into an useful number or the result of some standardized experiment“ [8], the most Ligand-based Virtual Screening(LBVS) methods use 2D fingerprint descriptors [9,10,11], then they calculate the similarity between molecules using similarity coefficients and some others methods. A query involves the specification of the entire structure of a molecule. The drug discovery process faces some challenges and some difficulties, such as complexity, cost and its time-consuming nature. Some studies estimate that the process, and the time and cost of discovering and developing a new drug takes over 10 years and costs approximately $1 billion [12]. To solve the high cost and reduce the time of developing drugs, a lot of researches are being done to provide methods and techniques that could contribute to easing the process. These studies have focused on aiding the efforts of the drug discovery process by using several computational methods. In this paper, we focused on the ligand-based virtual screening (LBVS) approach, which is based on comparative molecular similarity and the analysis of compounds that have known and unknown activities. This approach involves using large molecular databases to find similar molecules that have similar biological properties, as the molecular similarity principle states that “similar compounds tend to have similar properties and activities” [13], and that means molecules that are structurally similar tend to have similar activities. This could be used to identify, analyse and predict the most biologically active compounds and then demonstrate the correlated structural features and chemical properties of molecules with specific activities.

In LBVS, many methods and techniques have been proposed [14,15,16] for instance, Zheng *et al.* studied the performance of twenty different LBVS methods and they found that the LBVS is a good predictive of lead identification [16], and the work by Ripphausen *et al*. discussed the LBVS from different viewpoints by analyzing the information that have been provided form peer-reviewed publication [17]. Other research used the Bayesian inference network (BIN) and molecular fragments reweighing to enhance LBVS [14], and another recent work used the quantum based similarity measure (QBS) that provided a clear enhancements for LBVS. In addition, other works applied different fusion rules and techniques using the group fusion and similarity fusion, for instance, Willett [18] discussed and demonstrated most of these fusion rules that available for similarity ranking, Ali and *et al.* [15] developed a Condorcet fusion which combines the outputs of similarity searches using different distance similarity coefficients. However, more research needs to be done to provide better results for LBVS methods.

The rest of the paper is organized as follows: the next section describes the general concepts of similarity searching. The third section describes the proposed method, the fourth section discusses the experimental design and the results that have been carried out, and the last section concludes the work.

## 2. Related Work

The similarity measures have been used in different aspects of sciences and for different purposes such as clustering, classification and retrieval problems, the way of finding the appropriate similarity measures for specific domain needs considerable efforts, for chemical area a lot of some many similarity measures have been proposed as earlier we explained, the researchers found that some of these similarity measures which are appropriately work with text and document retrieving also properly work with chemical information [19], for this reason in chemoinformatics researchers give high consideration to the similarity measures proposed in the document and text retrieval area, one of these recently proposed similarity measures for the document retrieval area is the A Similarity Measure for Text Classification and Clustering (SMTP) that proposed by Lin *et al*. [20], which gained interest in this work, as the SMTP provided a good solution for document classification and clustering based on the flowing similarity proprieties [20]:

1. The presence or absence of a feature is more essential than the difference between the two values associated with a present feature.
2. The similarity degree should increase when the difference between two non-zero values of a specific feature decreases.
3. The similarity degree should decrease when the number of presence-absence features increases.
4. Two documents are less similar to each other if none of the features have non-zero values in both documents.
5. The similarity measure should be symmetric.
6. The value distribution of a feature is considered, *i.e.*, the standard deviation of the feature is taken into account, for its contribution to the similarity between two documents. A feature with a larger spread offers more contribution to the similarity between d_1_ and d_2_.

SMTP is used to measure the similarity measures between two documents, and also it has been extended to measure the similarity between two sets of documents. The SMTP has been derived from different similarity measures that have been used for text such as Dice, Euclidean, Extended Jacard, Cosine which are shown in Table 1 where d_1_ and d_2_ are two documents represented as vectors.

The SMTP measure the similarity between two documents $d_{1}\;=\;\left\{d_{11},\;d_{12}\ldots \;d_{1m}\right\}\;$ and $d_{2}\;=\;\left\{d_{21},\;d_{22}.\;.\;.\;d_{2m}\right\}\;$. Define a function *F* as follows in Equation (1) 1below:

(1) $$F\left(d_{1},d_{2}\right)=\frac{\sum_{j=1\;}^{m}N*\left(d_{1j\;},\;d_{2j}\;\right)\;}{\sum_{j=1\;}^{m}N\cup^{\text{​}}\left(d_{1j\;},\;d_{2j}\;\right)\;}$$

where this function numerator and denominator have different cases according to the compared features values as mentioned below.

$N*\left(d_{1j},d_{2j}\right)$ has three different cases according the value of compared features:

1. $N*\left(d_{1j},d_{2j}\right)=0.5\left(1+exp\left\{-{\left(\frac{d_{1j}-d_{2j}}{\sigma_{j}}\right)}^{2}\right\}\right)\;\mathrm{if}\;(d_{1j}\;\times \;d_{2j})>0$.
2. $N*\left(d_{1j},d_{2j}\right)=0\;\mathrm{if}\;d_{1j}=0\;\mathrm{and}\;d_{2j}=0\;$.
3. $N*\left(d_{1j},d_{2j}\right)=\;-\lambda \;,\;\mathrm{otherwise}$

$N\cup^{\text{​}}\left(d_{1j},d_{2j}\right)\;$ has two cases:

1. $N\cup^{\text{​}}\left(d_{1j},d_{2j}\right)=0,\;\mathrm{if}\;d_{1j}=0\;\mathrm{and}\;d_{2j}=0;\;$
2. $N\cup^{\text{​}}\left(d_{1j},d_{2j}\right)=1,\;\mathrm{otherwise};\;$

where: $d_{1},\;d_{2}$: document 1 and document 2 (compared document)

*J*: number of features (vectors).
σ: standard deviation of all non-zero values in a vector.
λ : small value of λ, e.g., 0.01–0.0001

Their proposed similarity measure as shown in Equation (2) below:

(2) $$S_{SMTP}\left(d_{1},d_{2}\right)=\frac{F\left(d_{1},\;d_{2}\right)+\lambda }{1+\lambda }$$

The measures of SMTP that are shown in Equation (2), are focused to meet all proprieties which provide solution of weakness of text similarity measures which are mentioned in Table 1, for the Euclidean does not meet properties 1, 3, 4, and 6 which are previously stated, and Cosine, Pairwise-adaptive, Extended Jaccard, Dice, and IT-Sim does not satisfy one or more of properties 3, 4 and 6. SMTP provides good results in classification and clustering. In this work we adopted SMTP to be used in ligand-based virtual screening.

## 3. Similarity Searching

The similarity between any two objects regardless of what that object is can be described as the degree of overlap between characteristic features of these specific objects. These overlaps are calculated using different ways and methods. In cheminformatics, the calculation of similarity measures in order to find matching molecules is not an easy task, as the molecular data representation is quite different from other data representations. For LBVS the similarity searching is considered as an important technique for screening chemical databases in order to identify those molecules that are most similar to other user-defined reference structures using computerized methods and techniques. In virtual screening, there are three main searching methods that are used for compound databases: the structure search, the substructure search and the similarity search. Structure searching focuses on searching a molecular database to check if there are presences or absences of a specific molecule in the database [21,22,23]. The substructure search focuses on the retrieval of molecules that contain a partial structure of a user-defined query [24,25]. Finally, similarity searching is focuses on searching the database to find a similar molecules by look for all the structures in a database that are achieving the highly similar to a given structure [26] for the exact match of molecules and concentrating on the specification of the entire structure of a molecule [27,28,29,30,31,32]. There are some works that combine more than research methods for screening [33]; in VS, the major task of similarity searching is to search databases to find which molecules in a database are similar or contain specific molecular structures and then detect fragments that are shared by the molecules. Many similarity coefficients have been applied in similarity searches and, for classification and clustering purposes, the literature is full of descriptions of these similarity coefficients. The work of Willett [26], and Todeschini *et al.* [34] discuss most of the proposed similarity measures in chemical databases. Other methods have also been used besides similarity coefficients, such as machine learning methods adapted for VS like the Naive Bayesian classifier [15,35,36], support vector machines (SVMs) [37,38] and voting techniques [37,39,40].

For the molecular similarity searching, many coefficients are introduced and applied with different fingerprint molecular databases. For instance, the Euclidian distance, Cosine, Dice, Forbes and Tanimoto coefficients, which is considered as the standard similarity measure for fingerprint-based similarity calculations [4].

## 4. Methods

### 4.1. Tanimoto Similarity Method

Both binary and distance similarity coefficients have been applied, as we mentioned before. A typical coefficient used in chemoinformatics is Jacard-Tanimoto, which is considered the most widely used asymmetric coefficient. It has two formulas for binary and continuous data, as shown in Equations (3) and (4) below, when the molecules *A* and *B* are represented by vectors, *x*, of length *N* with the number of property having the value $x_{i}$.

Equation (3) Tanimoto continuous variable formula:

(3) $$S_{A,B}=\frac{\sum_{i=1}^{N}X_{i}AX_{i}B}{\sum_{i=1}^{N}{\left(X_{i}A\right)}^{2}+\sum_{i=1}^{N}{\left(X_{i}B\right)}^{2}-\sum_{i=1}^{N}X_{i}AX_{i}B}$$

Equation (4) Tanimoto binary variable formula:

(4) $$S_{\;A,B}=\frac{c}{a+b-c}$$

*a* = bits set to 1 in A; *b* = bits set to 1 in B; *c* = number of 1 bits common to both.

Besides that, there are also other measures that have been popularly adopted for computing the similarity between two molecules derived for different areas and these have achieved good results.

### 4.2. The Adapted Similarity Measure of Text Processing (ASMTP)

The molecules are usually represented in fingerprints, which represent a way of encoding the structure of a molecule. The most common type of fingerprints is a series of binary digits (bits) that represents the presence or absence of particular substructures in the molecule. These features are stored in vectors where each component indicates the value of the corresponding feature in the molecule. The feature values are represented as bit-strings values. The investigation of these bit-strings values show that approximately 90% are zeros, and this sometimes causes difficulty and complexity when quantifying the similarity in drug discovery compared to the other domains of quantifying objects.

The proposed ASMTP algorithm has been derived for the text area, as we found that most of the algorithms developed for textual database processing can be used for processing chemical structure databases [1,19]. This has been applied in several text applications, including single label classification, multi-label classification, k-means like clustering and hierarchical agglomerative clustering, and the results obtained demonstrate the effectiveness of the proposed similarity measure. The documents and text databases are structured typically to the molecular databases, where both use small numbers for representing documents and chemical data by vectors. ASMTP is an adaptation of recently work of [20] which relies on three similarity properties concepts:

(a) The feature appears in both documents.
(b) The feature appears in only one document.
(c) The feature appears in none of the documents.

There are different assumptions for each of the mentioned proprieties concepts. For the first case, the assumption was that similarity increases as the difference between the two involved feature values decreases. For the second case they put an assumption that a fixed value contributed to the similarity. For the last case, if the feature does not appear in the compared objects, this will result in there being no contribution to the similarity. In addition to these similarity properties, the values of the distribution and average of the feature values are taken into account. This has a good effect on calculating the similarity between two molecules. The features with a larger spread make more contributions to the similarity between compared molecules.

For chemical databases we applied SMTP for ligand-based virtual screening by making some modifications to its equation as discussed below.

Suppose there is a molecule $m_{1},m_{2}$ with *j* features $f_{1},f_{2}$. Whereby *Fj* is represented as a *j*-dimensional vector. The proposed similarity measure ASMTP, which is based on the above mentioned similarity properties, has function *F* defined as in Equation (5) below:

(5) $$f\left(m_{1},m_{2}\right)=\frac{\sum_{j=1\;}^{m}N*\left(m_{1j}\;,m_{2j}\right)\;}{\sum_{j=1\;}^{m}N\cup^{\text{​}}\left(m_{1j},\;m_{2j}\right)}$$

where the numerator of *F* function$\;N*\left(\;m_{1j}\;,\;m_{2j}\right)$ has three different cases, according to the value of compared features as shown below in Equation (6):

(6) $$\begin{array}{l} 1.\;N*\left(m_{1j},m_{2j}\right)=0.5\left(1+exp\left\{-{\left(\frac{m_{1j}-m_{2j}}{\mu_{j}}\right)}^{2}\right\}\right)\;if\;(m_{1j}\times \;m_{2j})>0\;\\ 2.\;N*\left(m_{1j},m_{2j}\right)=0\;if\;m_{1j}=0\;and\;m_{2j}=0\;\\ 3.\;N*\left(m_{1j},m_{2j}\right)=-\lambda ,\text{ otherwise}.\; \end{array}$$

And $\mu =\frac{\sum_{i=1}^{n}m_{ij}}{n_{j}}$

The denominator of *F* function $N\cup^{\text{​}}\left(m_{1j},m_{2j}\right)$ has two different cases:

1. $N\cup^{\text{​}}\left(m_{1j},m_{2j}\right)=0,\;if\;m_{1j}=0\;and\;m_{2j}=0$.
2. $N\cup^{\text{​}}\left(m_{1j},m_{2j}\right)=1\;,\text{ otherwise}.\;$

The similarity measure equation of ASMTP is shown in Equation (7) below:

(7) $$S_{SMTP}\left(m_{1},m_{2}\right)=\frac{F\left(m_{1},m_{2}\right)+\lambda }{1+\lambda }$$

where:

$m_{1}$: Molecule1 (query).
$m_{2}$: Molecule 2(reference).
*J* = feature index (vector).
μ: is the average of all non-zero values of vector (feature) j.
λ: small value λ, e.g., 0.01–0.0001.
$n_{j}$_j_: total number of non-zero values in the features index.

The proposed ASMTP measure takes into account the following three cases: (a) The feature that appears in both compared molecules; (b) the feature that appears in only one of the compared molecules; and (c) the feature that appears in none of the compared molecules.

Each one of the mentioned cases has a different way of calculating the first case, so we set a lower bound 0.5 and decrease the similarity as the difference between the feature values of the compared molecules increases. This is scaled by a Gaussian function, as shown in Equation (5) where we have modified to use μ for representing the average of all non-zero values for a specific feature (feature vector) j in the molecules data set instead of σ which was representing the standard deviation of all non-zero values for feature in the training data in SMTP. In the second case, a negative constant −λ disregarding the magnitude of the non-zero feature value has been added. For the last case, when the feature has not appeared on both compared molecules, this is considered to have no effect on the similarity and no contribution to the similarity. For λ, it has taken a small value for our test using ASMTP, which is the value = 0.0001.

ASMTP is used to enhance the molecular ranking performance in VS by performing similarity calculations that improve the computational efficiency and help to rank and sort molecules in decreasing order of highest probability ratio. The aim is determine the result of the screening in the top 1% and 5%, so the ASMTP will simplify the calculation process and reduce the algorithm programming complexity.

## 5. Experimental Design

In this section, we investigate the effectiveness of the proposed similarity measure of ASMTP. The investigation is done by conducting several experiments of the simulated VS searches on two different benchmark datasets: the MDL Drug Data Report (MDDR) [41] and the maximum unbiased validation (MUV) [42]. It has been used widely for LBVS, and have also been used by our research group in some previous works [15,43]. All datasets contain 2D structural representations that have been converted to Pipeline Pilot’s ECFC_4 (Extended Connectivity) fingerprints and flooded to 1024 feature sizes [44], whereby the MDDR database contains 102,516 active and inactive molecules. From the MDDR we used two datasets: DS1 and DS2. The DS1 dataset contains 11 activity classes, containing structurally homogeneous and heterogeneous active classes. The DS2 dataset is different from DS1 as it contains more than 10 homogeneous activity classes. The details of selected activity classes of the MDDR are shown in Table 2 and Table 3. Each table contains an activity class index, the activity class name and the number of active molecules that belong to each class. The second dataset is the MUV, which is prepared for VS and has seventeen activity classes that have varying numbers of actives and decoys (Table 4).

## 6. Results and Discussion

The experiments were conducted by performing the proposed ASMTP algorithm. This simulated the VS search by using ten reference structures from each activity class randomly, as the selected references were unified and applied to Tanimoto and the ASMTP. The final output of obtained similarity results of the whole molecules of the database will then be ranked in decreasing order. The average retrieved output of the ten references’ query results mean are calculated in the 1% and 5% cutoffs of the recall data, while the procedure is repeated for the all databases. The common method of evaluating any similarity searching method is achieved by determining where the active compounds appear in the ranked list if the number of known active compounds listed in the top will be an indicator of the effective VS method. The effectiveness of the proposed ASMTP algorithm is evaluated using MUV and MDDR benchmark datasets.

The experiment results obtained by ASMTP, Tanimoto and one of the most recently VS similarity measure technique called Standard Quantum-Based(SQB) that have been proposed by Al-Dabbagh and *et al.* [43] are shown in Table 5, Table 6 and Table 7. Each table of results shows the database activity classes in the first column, while the second and third columns respectively show the average of recall of the ranking results for all activity classes at the cut off 1% and 5% for the standard similarity coefficient Tanimoto, and the fourth and fifth columns represent the SQB corresponding results and the seventh and eighth columns show ASMTP results. The end of each column shows the overall average recalls results of all classes. The best average recall for each class is highlighted. In the bottom of each column there is a shaded cells row that corresponds to the total number of shaded cells for the Tanimoto and the proposed method that achieved better results. The obtained results DS1 and DS2 are shown in (Table 5 and Table 6), and the results of MUV is shown in (Table 7).

The overall average results of all datasets achieved good results and outperformed Tanimoto and SQB. The DS1 achieved good results in six out of 11 classes in a cut off 1% and seven out of 11 in a cut off 5%. The DS2 achieved good results in nine out of 10 classes in a cut off 1%, and 10 out of 10 in cut off 5%. This is considered a good indicator of the high performance of the ASMTP algorithm. The MUV results also outperformed Tanimoto and SQB in both cut offs 1% and 5%, and 11 out of 17 MUV classes achieved good results in cut offs 1% and 5% respectively.

In addition, to examine the effectiveness of the proposed method ASMTP ,we used some of the most widely used evaluation method for VS, firstly we applied the Receiver Operating Characteristic (ROC) curve which has been used in various fields (medicine, meteorology, *etc.*) and also in the drug discovery field [45,46]. A ROC curve describes the tradeoff between sensitivity and specificity, which are the main characteristic features of any test. In the drug design context, sensitivity (Se) would be the percentage of truly active compounds being selected from the virtual screening workflow: the number of true positive (TP) results divided by the sum of true positives and false negatives (FN):

(8) $$\text{Sensitivity}=\frac{\text{Number selected actives}}{\text{Number of total actives }}\;=\;\frac{TP}{TP+FN}$$

The Specificity (Sp) represents the percentage of truly inactive compounds being correctly identified by the computer test and therefore being discarded, that is, the number of true negative results (TN) divided by the sum of true negatives and false positives (FP):

(9) $$\text{Specificity}=\frac{\text{Number of discarded inactives}}{\text{Number of inactives}}\;=\;\frac{TN}{TN+FP}$$

**Figure 1:**
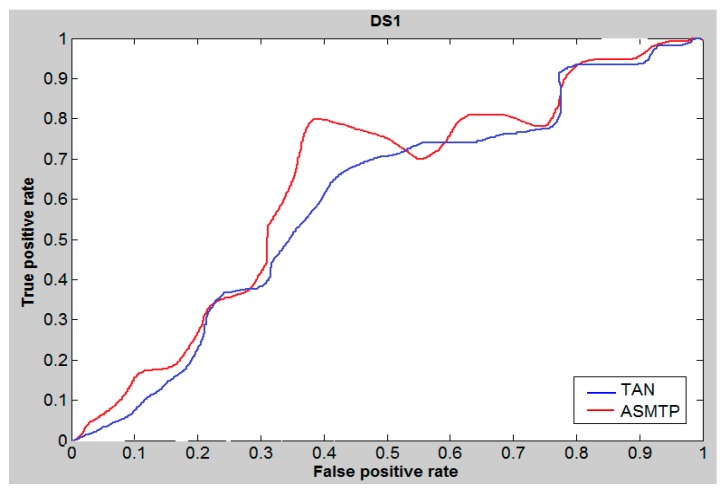
ROC curves and AUCs at 5% cutoff of DS1 data set.

**Figure 2:**
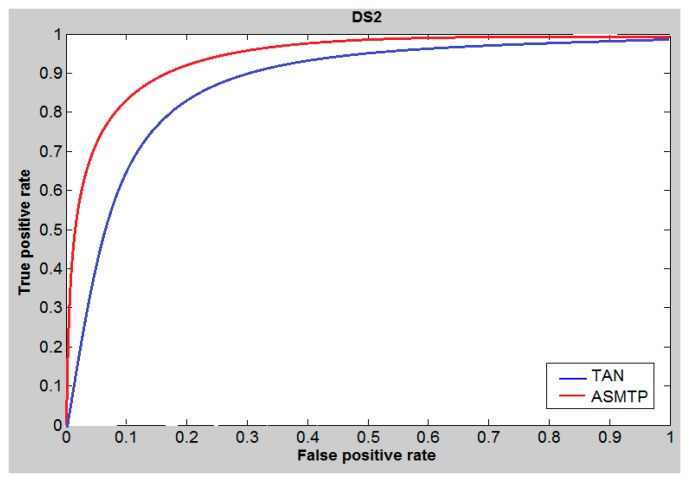
ROC curves and AUCs at 5% cutoff of DS2 data set.

**Figure 3:**
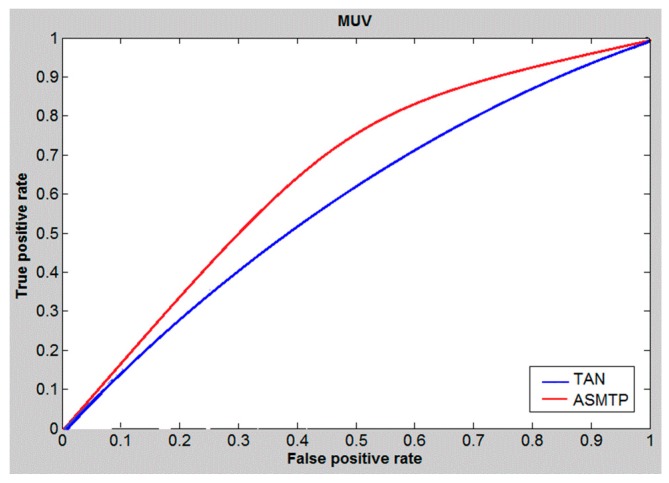
ROC curves and AUCs at 5% cutoff of MUV data set.

The area under the ROC curve (AUC) is a measure used to measure the test performance for the he closer AUC is to 1, the better is the performance of the prediction. For ASMTP we applied the ROC curve to study and evaluate the performance of the proposed ASMTP at cutoff 5%. Figure 1, Figure 2 and Figure 3, illustrate and provides a preliminary indication about the quality of the proposed method for data set DS1, DS2, and MUV compared to conventional similarity measures Tanimoto, and we can say that the conclusion derived from these tables (Table 5, Table 6 and Table 7) provides the same conclusion that derived from Figure 1, Figure 2 and Figure 3 that confirms the superior of ASMTP method.

Beside ROC curve, we also used Boltzmann enhanced discrimination of receiver operating characteristic (BEDROC) [45] which is based on the idea of exponentially weighted active ranks. And it is a little bit different from the enrichment factor (EF) or the receiver operating characteristic (ROC), for it concentrates on the beginning of a ranked list, and giving more weight to the compounds that will be ranked early the BEDROC scores are bounded between 0 and 1, where higher scores indicating more known actives being ranked on earlier ranks. Table 8, shows the results of EF and the BEDROC, The conclusion which can be drawn from all above evaluation methods confirm that ASMTP search outperformed the conventional similarity measure and SQB performance or at least it is not worse than them.

In spite of the recent work done by Nagwani [47], where he reported some limitations of SMTP in document retrieving. Conversely, by applying the ASMTP in LBVS we found that it works properly, and it has achieved good results which prove that the ASMTP is working properly with both datasets that contain most similar molecules (homogeneous) and also it enhanced the retrieval performance of diversity data (heterogeneous) data sets.

## 7. Conclusions

In this study, we presented a new LBVS similarity measure that has been derived from the document and text searching areas. The adapted ASMTP algorithm focuses on the preferred selected similarity properties, we conduct the experiments on two benchmark datasets the MDDR and MUV, and compared the achieved results with the Tanimoto coefficient which is considered as the conventional similarity measure in VS, and also we compared the results with QSB which is most recently proposed similarity measure for LBVS , We also have investigated the effectiveness of ASMTP performances by applying a set of VS mostly used evaluation methods. The overall achieved results from conducting screening and evaluation results show that the performance obtained by the proposed measure is improved LBVS with heterogeneous molecules data, and achieved superior results with data that are structurally homogeneous.

## Figures and Tables

**Table 1:** Similarity measures most used in text retrieval.

Similarity Measure	Formula
Extended Jaccard coefficient	S${\;}_{EJ}\left(d_{1}\;,d_{2}\right)\;$= $\frac{\left(d_{1\;}\;\times \;d_{2}\right)\;}{\;\left(d_{1}\;\times \;d_{1}\right)+\;\left(d_{2}\;\times \;d_{2}\right)\;–\;\left(d_{1}\times \;d_{2}\right)\;}$
Dice	$s_{Dic}\left(d_{1}\;,d_{2}\right)\;$=$\;\frac{2d_{1}\;\times \;d_{2}}{\left(d_{1}\times \;d_{1\;})\;+(d_{2}\;\times \;d_{1\;}\right)}$
Euclidean distance	$d_{EUC}$ $\left(d_{1}\;,d_{2}\right)\;=$ $\sqrt{(d_{1}-d_{2})\times \left(\;d_{1}-d_{2}\right)}$
Cosine similarity	$s_{Cos}\left(d_{1}\;,d_{2}\right)=\;\frac{d_{1\;}\;\times \;d_{2}}{\sqrt{d_{1}\;\times \;d_{1}}\;\sqrt{d_{2}\;\times \;d_{2}}}$
Pairwise-adaptive	$d_{Pair}=\left(d_{1}\;,d_{2}\right)\;$=$\;\frac{d_{1,k\;}\;\times \;d_{2},k}{\sqrt{d_{\left(1,k\right)}\;\times \;d_{\left(1,k\right)}}\;\sqrt{d_{\left(2,k\right)}\;\times \;d_{\left(2,k\right)}}}$
IT-Sim	$S_{it}\left(d_{1}\;,d_{2}\right)=\frac{2\sum_{wi}\min\left(p_{1i\;,\;}p_{2i\;}\right)\log\pi \left(w_{i}\right)}{\sum_{wi}p_{1i}\log\pi \left(w_{i}\right)\;+\;\sum_{wi}p_{2i}\log\pi \left(w_{i}\right)}$

where d*_i__,__K_* in Pairwise-adaptive is a subset of $d_{i},\;i\;=\;1,\;2,$ containing the values of the features which are the union of the *K* largest features appearing in d_1_ and d_2_, respectively.

**Table 2:** MDDR activity classes for the DS1 dataset.

Activity Index	Activity Class	Active Molecules
31420	Renin inhibitors	1130
71523	HIV protease	750
37110	Thrombin inhibitors	803
31432	Angiotensin II AT1antagonists	943
42731	Substance P antagonists	1246
06233	Substance P antagonists	752
06245	5HT reuptake inhibitors	359
07701	D2 antagonists	395
06235	5HT1A agonists	827
78374	Protein kinase C inhibitors	453

**Table 3:** DS2 dataset activity classes.

Activity Index	Activity Class	Active Molecules
07707	Adenosine (AI) agonists	207
07708	Adenosine (A2) agonists	156
31420	Rennin inhibitors 1	1300
42710	CCK agonists	111
64100	Monocycle_ lactams	1346
64200	Cephalosporin’s	113
64220	Carbacephems	1051
64500	Carbapenems	126
64350	Tribactams	388
75755	Vitamin D analogues	455

**Table 4:** MUV activity classes.

Activity Index	Activity Class
466	S1P1 rec. (agonists)
548	PKA (inhibitors)
600	SF1 (inhibitors)
644	Rho-Kinase2 (inhibitors)
652	HIV RT-RNase (inhibitors)
689	Eph rec. A4 (inhibitors)
692	SF1 (agonists)
712	HSP 90 (inhibitors) 30
713	ER-a-Coact. Bind. (inhibitors)
733	ER-b-Coact. Bind. (inhibitors)
737	ER-a-Coact. Bind. (potentiators)
810	FAK (inhibitors
832	Cathepsin G (inhibitors)
846	FXIa (inhibitors)
852	FXIIa (inhibitors)
858	D1 rec. (allosteric modulators)
859	M1 rec. (allosteric inhibitors)

**Table 5:** The recall is calculated using the top 1% and top 5% of the DS1 dataset.

Activity Classes	TAN		SQB		ASMTP	
	1%	5%	1%	5%	1%	5%
31420	57.3	85.85	73.73	87.22	78.83	96.81
71523	29.96	58.09	26.84	48.7	12.82	51.94
37110	14.38	29.98	24.73	45.62	39.53	63.84
31432	36.37	76.85	36.66	70.44	45.22	97.45
42731	16.89	27.74	21.17	19.35	13.95	20.88
6233	22.72	37.78	12.49	21.04	22.77	36.75
6245	5.03	14.83	6.03	13.63	11.73	26.26
7701	8.45	23.07	11.35	21.85	8.95	17.26
6235	9.03	21	10.15	19.13	21.91	37.17
78374	12.08	17.81	13.08	20.55	1.77	2.65
78331	8.77	16.71	5.92	13.1	3.31	10.24
Mean	20.08909	37.24636	22.01364	34.05	23.65	41.93182
Shaded cells	3	4	0	0	8	7

**Table 6:** The recall is calculated using the top 1% and top 5% of the DS2 dataset.

Activity Classes	TAN		SQB		Proposed Method	
	1%	5%	1%	5%	1%	5%
09249	61.84	70.39	58.5	74.22	72.82	73.3
12455	47.03	56.58	55.61	100	99.35	100
12464	65.1	88.19	62.22	95.24	81.66	96.46
31281	81.82	86.64	83	93	92.73	99.09
43210	80.31	93.75	80.73	98.94	88.2	99.85
71522	53.84	77.68	53.13	98.93	81.25	99.11
75721	46.8	63.94	34.61	90.9	77.27	98.67
78331	30.56	44.8	29.04	92.72	80	96.8
78348	80.18	91.71	81.86	93.75	82.17	99.74
78351	87.56	94.82	85.4	95.39	96.48	96.92
Mean	63.504	76.85	62.41	93.31	85.193	95.994
Shaded cells	0	0	0	0	10	10

**Table 7:** The recall is calculated using the top 1% and top 5% of the MUV 17 activity classes data sets.

Activity Index	Tanimoto		SQB		Proposed Method	
	1%	5%	1%	5%	1%	5%
466	3.1	5.86	1.38	8.62	5.86	9.66
548	8.62	22.76	11.38	24.14	10.34	17.93
600	3.79	11.38	5.52	16.21	6.21	13.45
644	7.59	17.59	8.97	17.93	7.24	12.41
652	2.76	7.93	3.79	9.66	5.86	11.38
689	3.79	9.66	4.48	11.72	5.86	9.71
692	0.69	4.83	1.38	4.83	3.79	6.55
712	4.14	10.34	5.17	11.03	6.21	8.97
713	3.1	7.24	2.76	5.86	6.21	9.31
733	3.45	8.97	4.14	8.62	5.86	9.31
737	2.41	8.28	1.72	8.28	7.59	14.14
810	2.07	6.9	1.72	11.03	7.24	13.1
832	6.55	13.1	8.28	14.83	13.1	20
846	9.66	28.62	12.41	26.9	10.69	25.52
852	12.41	21.38	9.66	20	13.45	21.03
858	1.72	5.86	1.38	6.21	6.21	7.93
859	1.38	8.97	2.41	8.62	5.86	10.69
Avg	4.54	11.70	5.09	12.61	7.50	12.991
Shaded cells	0	2	3	5	14	11

**Table 8:** Comparison results of enrichment values of (BEDROC α = 20) and (EF 1%) using ASMTP on MDDR1, MDDR2, and MUV data sets.

Methods	DS1				DS2				MUV			
	BEDROC( α 20)		EF (1%)		BEDROC( α 20)		EF (1%)		BEDROC( α 20)		EF (1%)	
	Mean	Median	Mean	Median	Mean	Median	Mean	Median	Mean	Median	Mean	Median
Tan	0.48	0.46	80.01	86.01	0.33	0.34	23.01	23.01	0.37	0.37	16.69	17.92
SQB	0.53	0.57	90.01	89.31	0.44	0.39	29.01	22.01	0.41	0.39	18.01	19.74
ASMTP	0.61	0.64	92.9	90.23	0.46	0.50	28.27	25.32	0.44	0.42	18.93	20.14
